# Multi‐Omic Analysis Reveals Population Differentiation and Signatures of Social Evolution in *Tetragonula* Stingless Bees

**DOI:** 10.1111/mec.17823

**Published:** 2025-06-11

**Authors:** Benjamin A. Taylor, Garett P. Slater, Eckart Stolle, James Dorey, Gabriele Buchmann, Benjamin P. Oldroyd, Rosalyn Gloag, Brock A. Harpur

**Affiliations:** ^1^ Department of Entomology Purdue University West Lafayette Indiana USA; ^2^ US Department of Agriculture Agricultural Research Service (USDA‐ARS) Baton Rouge Louisiana USA; ^3^ Leibniz Institute for the Analysis of Biodiversity Change (LIB), Museum Koenig Bonn Germany; ^4^ Environmental Futures University of Wollongong Wollongong New South Wales Australia; ^5^ Behaviour, Ecology and Evolution Laboratory, School of Life and Environmental Sciences A12 University of Sydney Sydney New South Wales Australia

**Keywords:** behaviour/social evolution, insects, major transitions, sociogenomics, transcriptomics

## Abstract

Stingless bees in the genus *Tetragonula* are social insects with a fully sterile worker caste, and are therefore well‐placed to provide insights into the genomic changes associated with ‘superorganismal’ life histories. Here we assemble the genome of *Tetragonula carbonaria* and characterise the population structure and divergence of both *T. carbonaria* and its cryptic congener *T. hockingsi* in eastern Australia, revealing three distinct populations for *T. carbonaria* and two partially differentiated subpopulations for *T. hockingsi* . We then combine our genomic results with RNA‐seq data from different *T. carbonaria* castes (queens, males, workers) to test two hypotheses about genomic adaptations in social insects: the ‘Relaxed Constraint’ hypothesis, which predicts indirect, and therefore relaxed, selection on worker‐biased genes; and the ‘Adapted Worker’ hypothesis, which predicts intensified positive selection on worker genes due to their evolutionarily novel functions. Although we do not find a direct signal of either weaker purifying selection or elevated positive selection in worker‐biased genes based on deviations from neutral expectations of nucleotide change between the two species, other evidence does support a model of relaxed selection on worker‐biased genes: such genes show higher nucleotide diversity and greater interspecies divergence than queen‐biased genes. We also find that differentially caste‐biased genes exhibit distinct patterns of length, GC content and evolutionary origin. These findings, which converge with patterns found in other social insects, support the hypothesis that social evolution produces distinct signatures in the genome. Overall, *Tetragonula* bees emerge as a valuable model for studying the genomic basis of social complexity in insects.

## 1 Introduction

Social insects—those species whose colonies are composed of one or a few reproductive individuals aided by a much larger cohort of non‐reproductive workers—are central models for understanding the evolution of cooperative living and its associated genomic signatures (Robinson et al. 2005; Smith et al. 2008; Yan et al. 2014; Toth and Rehan 2017; Jones et al. 2023). Of special interest are ‘superorganismal’ species, the subset of social insect species in which adult workers are developmentally committed to a sterile or subfertile role and can therefore never inherit the nest and become queens. In these species, the reproductive caste tends to express conserved phenotypes such as egg‐laying and mate‐finding, while workers may exhibit derived phenotypes such as extremely specialised morphology and highly altruistic nest defence behaviour (Jaffé et al. 2007; Shorter and Rueppell 2012; Gordon et al. 2019). Superorganismal species can be described as having undergone a major evolutionary transition: a fundamental shift in the level at which selection operates (Boomsma and Gawne 2018; Taylor et al. 2019; Boomsma 2022). This is because reproductive individuals (queens and males) in such species are exposed directly to selection, while workers' traits are either primarily or solely selected indirectly via the effects of their altruistic behaviour.

Caste‐specific modes of selection are predicted to produce unique molecular signatures in the genomes of superorganismal social insects (Mikhailova et al. 2024). One specific set of predictions is derived from the fact that, because genes in sterile workers are selected only indirectly (via their influence on the queen's direct reproduction) these genes are expected to experience weaker selection compared to genes that are expressed in reproductive individuals (Crozier and Pamilo 1996). It follows that genes whose functions are primarily expressed in such sterile workers should exhibit signatures of relaxed purifying selection (Linksvayer and Wade 2009). This relaxed constraint is predicted to result in worker‐expressed genes having both higher polymorphism within populations, and higher divergence between populations, than genes under direct selection. This is because weakly deleterious alleles are more likely to persist, and to drift to fixation, when purifying selection is weak. Hereafter, we refer to this model as the ‘Relaxed Constraint’ hypothesis of worker evolution.

A second set of predictions regarding the molecular signatures of caste‐associated genes derives from the fact that the derived features (relative to non‐social ancestors) that are associated with superorganismal group living are disproportionately expressed in workers rather than reproductives. These derived phenotypes include obligate worker subfertility (Boomsma and Gawne 2018; Bernadou et al. 2021), alloparental care (Chouvenc 2022) and ‘social hygiene’ behaviours (Pull and McMahon 2020), as well as lineage‐specific traits such as the honey bee waggle dance (von Frisch 1967) or the hyperspecialised soldier subcastes of some ant and termite species (Jaffé et al. 2007; Gordon et al. 2019; Miura and Maekawa 2020). Since these evolutionarily novel traits are expressed primarily by workers, it is possible that genes with worker‐biased expression might exhibit elevated signatures of positive selection (Harpur et al. 2014), a prediction that we hereafter refer to as the ‘Adapted Worker hypothesis’ (following Harrison et al. 2021).

While both the Relaxed Constraint and Adapted Worker hypotheses are theoretically compelling, attempts to empirically assess the predictions of these hypotheses (that worker‐expressed genes should exhibit elevated signatures of relaxed selection and positive selection respectively) have not converged upon a clear result (reviewed in Mikhailova et al. 2024). In favour of the Adapted Worker hypothesis, worker‐biased genes have been found to exhibit elevated signatures of positive selection in studies of honey bees (Harpur et al. 2014) and of at least one termite species (Radford et al. 2022). However, a separate study of four superorganismal termite species found little evidence for differences in signatures of selection among caste‐biased genes, with the majority of caste‐biased genes in these species instead showing signatures of strong purifying selection (Harrison et al. 2021). Seemingly in favour of the Relaxed Constraint model, Hunt et al. (2010) and Lucas et al. (2017) found that, in the superorganismal ants *Solenopsis invicta* and *Laisus niger*, caste‐biased genes exhibited signatures of relaxed selection. However, this was true regardless of whether the bias was in favour of queens and workers; moreover, Hunt et al. (2010) found that those same genes also exhibited signatures of relaxed selection in non‐social lineages, which might instead indicate that genes under relaxed selection are more likely to become caste‐biased, rather than caste‐biased genes secondarily becoming subject to relaxed selection. Given the variability in the results of these studies, and the fact that these results have been derived using only a very small number of independent lineages, data from additional superorganismal taxa are needed if we are to assess the consistency of genomic changes associated with social living.

Stingless bees (Apidae, Tribe Meliponini) comprise more than 500 species distributed across the global tropics and subtropics (Grüter 2020). They, when grouped with bumble bees, represent one of the two independent origins of superorganismal sociality within the Apoidea (the other being honey bees, *Apis* spp.; Cardinal and Danforth 2011; da Silva 2021). In all honey bees and some stingless bees, workers can lay unfertilised (male) eggs (e.g., honey bee workers do so in the absence of a queen; Winston 1991) and are therefore not entirely sterile, such that selection, while mostly indirect, can act directly on some traits in workers. Workers in the Australian stingless bee *Tetragonula carbonaria* , however, are obligately and irreversibly sterile (Bueno et al. 2020), such that they never reproduce, even when the colony becomes queenless (Gloag et al. 2007; Nunes et al. 2015). *Tetragonula* thus represents a superorganismal lineage in which selection on worker‐expressed genes is exclusively indirect.

**FIGURE 1:**
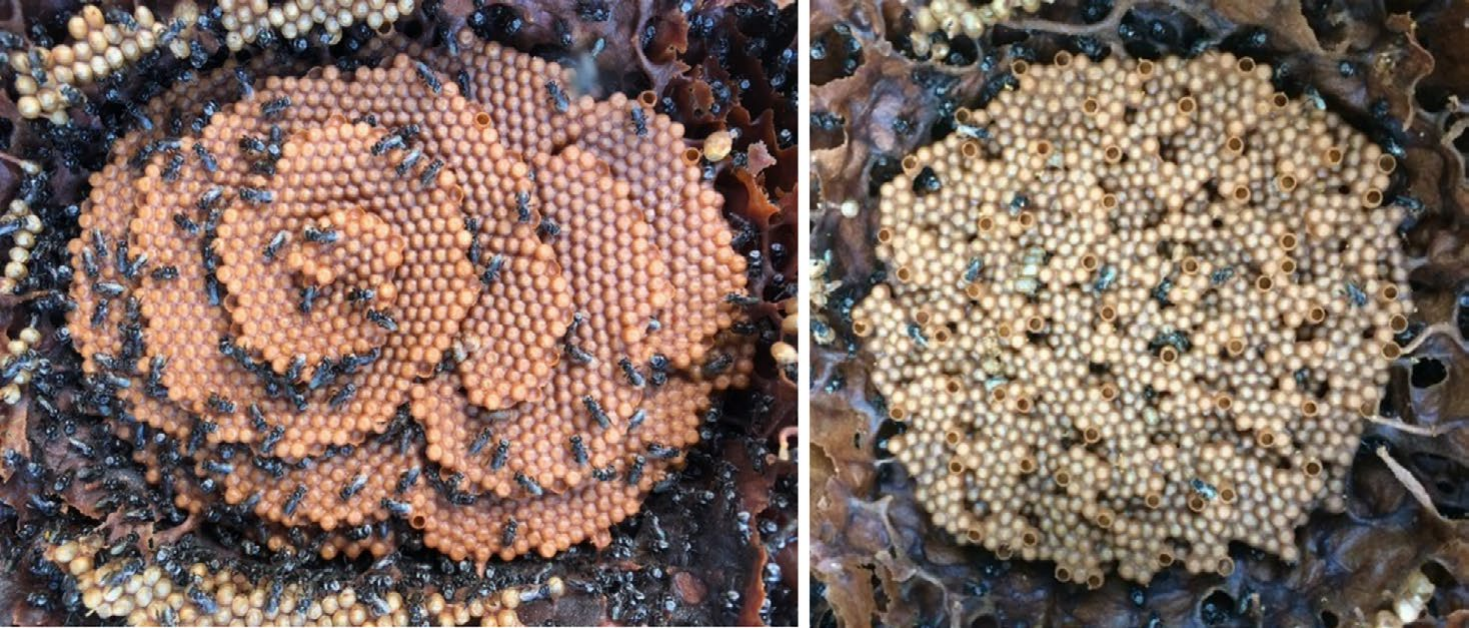
Nest architecture of the stingless bee *Tetragonula carbonaria* (left) and its cryptic congener *T. hockingsi* (right).

Both *T. carbonaria* and its closely related congener *T. hockingsi* are broadly distributed along the tropical and subtropical east coast of Australia, with some regions of sympatry. The two species are morphologically cryptic: workers of *T. hockingsi* are marginally larger on average than those of *T. carbonaria* , but the size distributions of each species overlap, and workers are otherwise identical in appearance (Dollin et al. 1997; Brito et al. 2014). Likewise, queens and males are indistinguishable between species, including at the level of male genitalia (Paul et al. 2023). Despite this, the two have long been recognised as separate species due to immediately recognisable differences in the architecture of their nests: *T. carbonaria* builds a highly distinctive spiral brood comb, whereas the brood cells of *T. hockingsi* are clustered (Figure 1; Dollin et al. 1997; Franck et al. 2004). This striking difference in brood structure likely arises from variation in the behavioural rules used by workers to choose the position of new brood cells (Brito et al. 2012). The species divergence time between *T. carbonaria* and *T. hockingsi* has been estimated at around 0.50 million years ago (Ma) based on a single nuclear gene (Ef1‐alpha; Françoso et al. 2019). However, both species also have significant within‐species population structure across their large ranges (Brito et al. 2014; Franck et al. 2004; Law et al. 2024; Nacko 2023). These bees are also important pollinators of native plants and are increasingly used as managed pollinators of tropical fruit crops across Australia (Halcroft et al. 2013; Kendall et al. 2022; Tierney et al. 2023), meaning that there is a timely need for population genomic data and resources for these species.

In this study, we begin by assembling a *T. carbonaria* genome, the first such assembly for any *Tetragonula* species. We use this assembly as a reference against which to identify discrete populations of *T. carbonaria* and *T. hockingsi* from population genomic data, a necessary step prior to testing sociogenomic predictions about the between‐population divergence of caste‐biased genes. In addition to confirming population structure, we also take advantage of our dataset to characterise genomic diversity and differentiation between and within these economically important but cryptic species, and to estimate population divergence times. We then combine these data with a newly sequenced gene expression dataset derived from *T. carbonaria* queens, workers and males in order to test hypotheses related to the relaxed constraint and adapted worker models of social genome evolution.

## 2 Methods

### 2.1 *Tetragonula carbonaria* Genome Assembly and Annotation

In brief, we performed an initial 10× genome assembly using one *T. carbonaria* male (haploid) pupa from a colony sourced from the University of Sydney Apiary, Sydney, New South Wales in 2017, which was then refined using short‐read (Illumina) and long‐read (Nanopore) data from additional female pupae sampled separately from Brisbane, Queensland, Australia. We annotated the resulting assembly using transcriptome assemblies generated from RNA‐seq data (see below). Full details of genome sequencing and assembly are given in Supporting Information File 1.

### 2.2 Population Structure: Diversity and Divergence Between and Within Species

#### 2.2.1 Sample Collection, DNA Extraction and Sequencing

**FIGURE 2:**
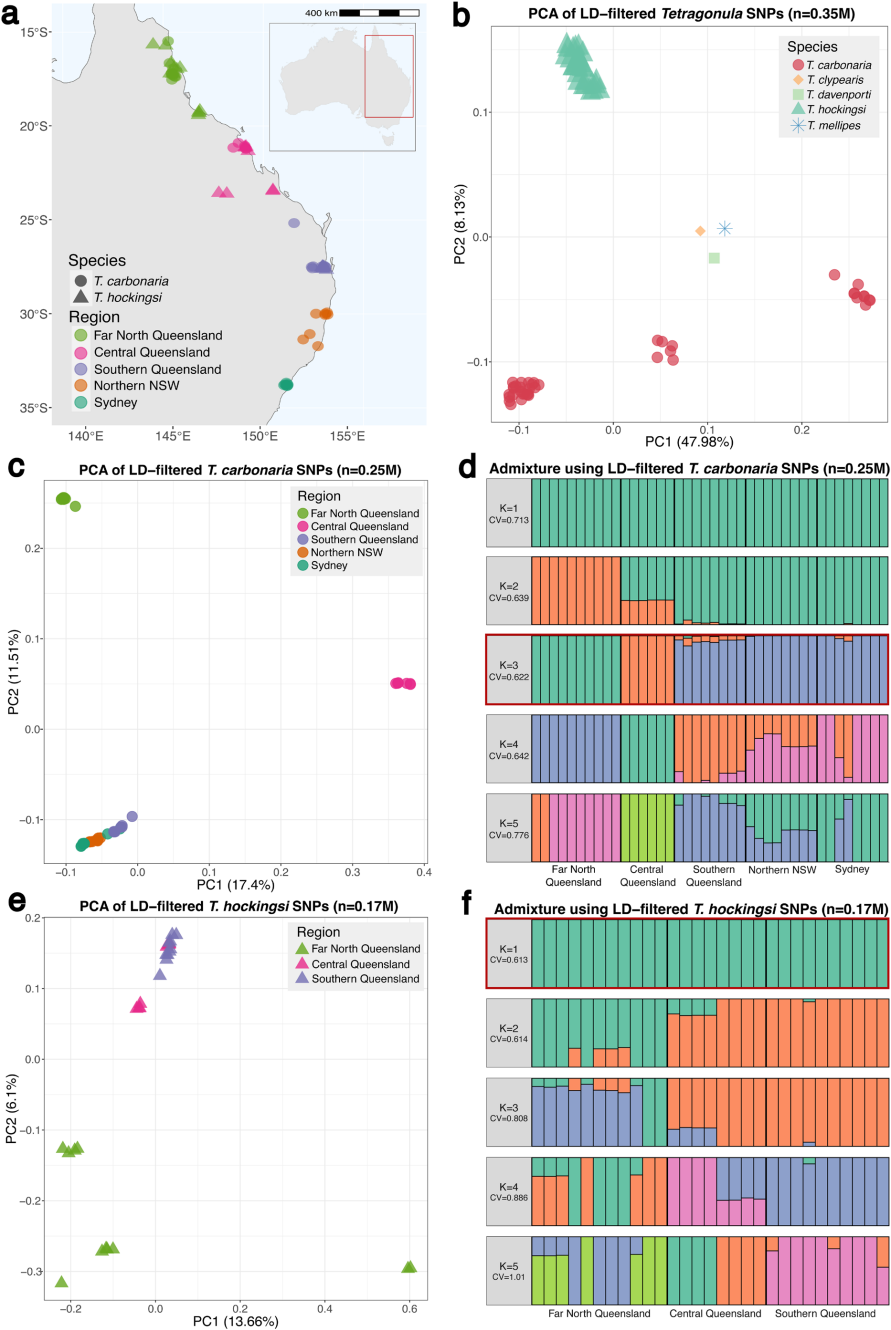
Population structure of *Tetragonula carbonaria* and *T. hockingsi* on Australia's east coast (a) Map of sample locations used for population genomic analyses. Inset: map of Australia showing sampling region in red. (b) Principal component analysis (PCA) of 0.35M linkage (LD)‐filtered SNPs for all samples. (c) PCA of 0.25M linkage‐filtered SNPs for all *T. carbonaria* samples. (d) Admixture analysis using 0.25M *T. carbonaria* linkage‐filtered SNPs. Optimal *K* value bordered in red. (e) PCA of 0.17M linkage‐filtered SNPs for all *T. hockingsi* samples. (f) Admixture analysis using 0.17M *T. hockingsi* linkage‐filtered SNPs. Optimal *K* value bordered in red.

We collected 70 female *Tetragonula* workers for population genetic analysis in 2018 and 2019 from localities across coastal Eastern Australia (Table S1). We chose localities corresponding to five broad regions (Figure 2a): Sydney (*N* = 8), Northern NSW (*N* = 8), Southern Queensland (*N* = 18), Central Queensland (*N* = 14), and Far North Queensland (*N* = 21). We sourced most samples from naturally occurring nests or locally sourced hives maintained by beekeepers (one per colony, *N* = 52). The remaining samples were foragers collected from flowers, with collection sites at least 1 km apart. Species was determined based on sequence similarity to samples of known species. In all, our dataset included 39 *T. hockingsi* and 30 *T. carbonaria* ; one sample from Far North Queensland (SRR25944842) was assigned to another species ( *Tetragonula clypearis* ) and excluded from further analysis.

We extracted DNA from whole bees using a Qiagen DNeasy Blood & Tissue Kit (ID: 69506). Genome sequencing was carried out at the Australian Genome Research Facility (AGRF) using Illumina HiSeqX to generate 150 bp pair‐end reads (mean ± SD coverage: 31.1 ± 6.0×). In addition to the newly sequenced samples described above, we also used existing data for an additional three samples representing other Australian *Tetragonula* (Hereward et al. 2020): *T. clypearis* (SRR10411628), *T. mellipes* (SRR10426324), and *T. davenporti* (SRR10405219). We removed low‐quality sequences using Trimmomatic v0.39 (Bolger et al. 2014) and then aligned reads to our newly created *T. carbonaria* reference genome using NextGenMap v0.5.2 with default parameters. We then used GATK v4.2.2.0 to remove duplicated reads and identify and filter SNPs using the following filters: QD < 2.0; QUAL < 30.0; SOR > 3.0; FS > 60.0; MQ < 40.0; MQRankSum < −12.5; ReadPosRankSum < −8.0. We retained filtered SNPs if they exceeded an allele count of 50 and a minor allele frequency of 0.05 across all samples, resulting in 0.84M SNPs among all samples; 0.69M among the *T. carbonaria* samples (*N* = 40); and 0.82M among the *T. hockingsi* samples (*N* = 29).

#### 2.2.2 Population Structure Within Species

To determine the structure of populations in each species across their range, we used admixture analysis (ADMIXTURE v1.3.0 with default parameters, *K* = 2–5; Alexander et al. 2009) and principal component analysis (PCA; snpgdsPCA function of the package *SNPRelate* v1.26.0 (Zheng et al. 2012) in R v4.1.2 (R Core Team 2022)). Prior to these analyses, we filtered SNPs for each focal species to exclude sites in strong linkage disequilibrium (LD) with one another (pairwise *r* ^2^ > 0.8 within sliding windows of 500 kb, 1 kb between windows; PLINK v2.0 (Chang et al. 2015)), retaining 0.35M SNPs among all samples; 0.25M for *T. carbonaria* ; and 0.17M for *T. hockingsi* .

#### 2.2.3 Within‐ and Between‐Population Patterns of Genetic Diversity

For each population identified using admixture and principal component analyses, we calculated nucleotide diversity (*π*) within 10,000 bp windows across the genome using VCFtools v0.0.16 (Danecek et al. 2011), with overall π taken as the mean of values across all windows. We calculated absolute nucleotide differentiation between pairs of populations (*dxy*) using pixy v1.2.6.b1 (Korunes and Samuk 2021). We calculated homozygosity (F_IS_) per individual (using VCFtools), with population values equal to the mean of values across all individuals in that population. We calculated Weir and Cockerham's (1984) *F*_ST_ estimator for each SNP between each pair of populations (VCFtools, —weir‐fst‐pop, default settings), with overall *F*_ST_ between populations being the mean of values across all SNPs (both weighted and unweighted means reported). To identify genes in each between‐population comparison with fixed or near‐fixed SNPs located in coding sequences, we took the subset of SNPs with *F*_ST_ > 0.95 and then used BedTools v2.29.0 (Quinlan and Hall 2010) to intersect these lists of SNPs with a bed file of CDS regions based on our *T. carbonaria* genome. We also assessed Tajima's D per population using VCFtools and between‐population patterns in signatures of past inbreeding, in the form of long runs of homozygosity (ROHs); further details for these metrics are given in Supporting Information File 1.

Additionally, to understand the extent to which genomic divergence between populations is driven by selection, we determined the proportion of SNPs exhibiting different values of relative population differentiation (*F*_ST_) between each pair of populations that were found in coding sequences (CDSes), with the hypothesis that highly differentiated SNPs would be disproportionately located within such sequences if population differences were principally driven by selection. To explore those genes that might be contributing to selectively driven population differences, we also identified genes which contained at least three highly differentiated CDS‐situated SNPs (*F*_ST_ > 0.90) for each between‐population comparison, and then performed GO enrichment analysis for these genes using the topGO package (Alexa and Rahnenführer 2009) in R. We used TopGO's weight01 algorithm and Fisher's exact tests to identify GO terms that were significantly overrepresented (*p* < 0.01) against a background consisting of all genes that appeared in the relevant analysis. We focused on biological process terms, but Supporting Information Tables include information for significant GO terms belonging to all ontologies.

#### 2.2.4 Population Divergence Times Estimates

Social bees in the family Apidae are among the handful of insect species for which there are direct estimates of the per‐generation spontaneous mutation rate (honey bees: 3.4 × 10^−9^ per haploid genome per generation; Yang et al. 2015; bumble bees: 3.6 × 10^−9^ per haploid genome per generation; Liu et al. 2017). We generated simple estimates of population divergence times within and between *T. carbonaria* and *T. hockingsi* populations by dividing the pairwise genetic distance (dxy) between each pair of populations by twice the mutation rate (assuming a mutation rate between 3.4 × 10^−9^ and 3.6 × 10^−9^).

### 2.3 *Transcriptomic* Analysis of Caste‐Biased Genes in *T. carbonaria*

#### 2.3.1 Sample Collection, RNA Extraction and Sequencing

We collected *Tetragonula carbonaria* samples for gene expression analysis from The University of Sydney Camperdown campus, with samples placed on dry ice and stored in RNAlater (Sigma‐Aldrich) at −80°C until RNA extraction.

We extracted RNA from three individuals from each of four groups (Table S2): (1) mature males (*n* = 3, collected from mating aggregations; Bueno et al. 2022), (2) mated, egg‐laying queens (*n* = 3, one each from three colonies), (3) foraging workers (collected returning to the nest; *n* = 3, one each from the same three colonies) and (4) within‐nest workers (collected by opening hives and sampling workers walking on the brood comb; *n* = 3, one each from the same three colonies). We extracted total RNA from abdomens and heads separately for each of these 12 samples using an RNeasy extraction kit (Qiagen) according to manufacturer instructions. Library preparation and sequencing of RNA extractions was performed by Novogene. mRNA was purified from total RNA using poly‐T oligo‐attached magnetic beads. After fragmentation, the first strand cDNA was synthesised using random hexamer primers followed by second strand cDNA synthesis. Then, 150 bp paired‐end sequencing to a depth of ~150M reads/sample was performed on a NovaSeq 6000 platform (Illumina) by Novogene.

#### 2.3.2 Gene Expression Quantification

We performed transcript filtering, trimming, alignment and quantification using the nf‐core/rnaseq pipeline v3.6 (Ewels et al. 2020; Patel et al. 2023) in Nextflow v21.10.3 (Di Tommaso et al. 2017). We used TrimGalore v0.6.6 (Krueger 2023) for adapter and quality trimming, followed by removal of ribosomal sequences using SortMeRNA v4.3.4 (Kopylova et al. 2012). We aligned reads to our recently assembled genome using STAR v2.6.1 (Dobin et al. 2013), with 85.24% (± 4.93%) reads successfully aligned, followed by quantification using Salmon v1.5.2 (Patro et al. 2017) and assembly of reads into genes using StringTie2 v2.1.7 (Kovaka et al. 2019).

#### 2.3.3 Differential Expression Analysis

We analysed differential expression in R using the DESeq2 package v1.42.1 (Love et al. 2014). Prior to this analysis, we filtered out genes with low expression: first, we removed genes whose mean expression across all samples was lower than 1 read/sample; second, we removed genes whose mean expression was below 5 reads/sample within all samples representing a unique combination of caste (male, queen, forager, in‐nest worker) and tissue (abdomen, head). We then identified differentially expressed genes (DEGs) between each pair of castes by specifying a combined DESeq2 model (~caste + tissue) with a parametric model fit. To identify genes differentially expressed only within a single tissue, we additionally considered individual models (~caste) using only samples belonging to each of the two focal tissue types. We considered that genes were differentially expressed in a given comparison if *p* < 0.05 against a baseline absolute log(2) fold change of 1 after false discovery rate correction (Benjamini and Hochberg 1995).

To obtain an equivalent dataset for the honey bee *Apis mellifera* as a comparison, we used honey bee allele frequency data obtained by Harpur et al. (2014) and RNA‐seq data generated from heads and abdomens of honey bee queens and workers by Warner et al. (2019), github.com/warnerm/devnetwork. These data were then subjected to the same quality filtering and DESeq2 model specification described above to generate lists of caste‐biased DEGs for *A. mellifera* . This allowed us to confirm that differences in the observed patterns for *Tetragonula* and those previously described for *Apis* were not due to differences in data analyses. We also identified reciprocal best hits between *T. carbonaria* and *A. mellifera* using BLAST+ 2.12.0 (Camacho et al. 2009) to compare orthologous genes across the two species.

#### 2.3.4 Tests of Relaxed Constraint and Adapted Worker Hypotheses

To determine whether there are associations between caste bias and gene features consistent with either the Relaxed Constraint or Adapted Worker hypotheses, we first generated consolidated lists of genes for each caste comparison by running an additive model in DESeq2 with *expression ~ caste + tissue* and then identifying DEGs within each pairwise caste contrast. We then assessed whether each set of DEGs exhibited differences in terms of three genomic attributes calculated using pixy v1.2.6.b1 (Korunes and Samuk 2021): (1) average per‐site heterozygosity (π) of the gene in *T. carbonaria* , (2) average number of nucleotide differences (*dxy*) between *T. carbonaria* and *T. hockingsi* , and (3) relative population differentiation (*F*_ST_). We also calculated Neutrality Index (NI; Rand and Kann 1996) and Direction of Selection (DoS; Stoletzki and Eyre‐Walker 2011) for the genes, again based on differences between *T. carbonaria* and *T. hockingsi* (all samples). Both NI and DoS were calculated using the proportion of synonymous and non‐synonymous substitutions and polymorphisms within the coding sequences of a given gene, which we acquired using SNPGenie v2019.10.31 (Nelson et al. 2015). If worker‐biased genes are under relaxed selection relative to queen‐biased genes, we hypothesised that such genes should exhibit elevated heterozygosity and NI, and reduced DoS, while these values should exhibit the opposite patterns under a model of intensified directional selection acting on worker genes. For *dxy* and *F*_ST_, bouts of recurrent selection, as predicted by the Adapted Worker hypothesis, are expected to result in elevated *F*_ST_ but not elevated *dxy*. We also calculated dN/dS (the ratio of substitution rates for nonsynonymous vs. synonymous codons) across each gene's coding sequences using PAML. Elevated dN/dS relative to the genomic background may occur as a result of positive selection or of relaxed selection, but the expected value of this metric varies under the two scenarios: under completely relaxed selection, dN/dS approaches a value of one, while values significantly above one are only expected to occur under positive selection.

Finally, because the Adapted Worker hypothesis partially stems from the idea that worker genes are evolutionarily labile, we also assessed the relative age of genes within each set of DEGs in two ways. First, we compared the effective length of genes, as computed by Salmon v1.5.2 (Patro et al. 2017), on the basis that shorter genes tend to have more recent evolutionary origins (Werner et al. 2018). Second, we performed phylostratigraphy in R using the phylostratr package v0.2.1 (Arendsee et al. 2019) to identify the putative evolutionary origin of each gene. We also calculated the % GC content of the gene using BedTools v2.29.0 (Quinlan and Hall 2010), as this correlates strongly with caste‐biased expression in honey bees (Kent et al. 2012; Harpur et al. 2014).

## 3 Results

### 3.1 *Tetragonula carbonaria* Genome Assembly and Annotation

Our final assembly consisted of 230 scaffolds with a total length of 298.7 Mbp, similar to genome size estimates of other recent stingless bee assemblies (283.99 Mbp for *Tetragonisca angustula* ; Ferrari et al. 2024; and 260.23 for *Melipona bicolor* ; Araujo et al. 2024). The N50 of the final assembly was 16.7 Mbp, with a maximum scaffold length of 24.8 Mbp. Of 5991 BUSCO groups, 98.3% were complete (98.1% both complete and single‐copy). Our final annotation contained models for 24,399 protein‐coding genes. Given that this number is considerably greater than equivalent estimates for other stingless bees (~17,500 for *T. angustula* and ~21,400 for *M. bicolor* ), it may be an overestimate. Indeed, when considering only genes for which at least one RNA‐seq read was identified, only 20,119 (82.5%) protein‐coding genes remained, in line with the numbers for other stingless bees.

### 3.2 Population Structure: Diversity and Divergence Between and Within Species

Both *T. carbonaria* and *T. hockingsi* exhibited substantial within‐species population structure. *T. carbonaria* samples formed three separate clusters in our principal component analyses (PCA), corresponding to populations in Far North Queensland (*N* = 10), Central Queensland (Mackay/Eungella region, *N* = 6) and a southern population stretching from Southern Queensland to Sydney (*N* = 24; Figure 1a–c). Admixture analyses similarly indicated three discrete *T. carbonaria* populations, with only minor evidence of admixture between samples from Central and Southern Queensland (Figure 2d); genomic differentiation between all three populations was high (*F*_ST_; Table 1).

**TABLE 1:** Genomic differentiation between each pair of populations and subpopulations.

Comparison	*F*_ST_ (mean)	*F*_ST_ (weighted)	*dxy*
*T. carbonaria* versus *T. hockingsi*	0.55653	0.66014	0.00456
*T. carbonaria* North versus South	0.37919	0.49323	0.00361
*T. carbonaria* North versus Central	0.35358	0.49846	0.00359
*T. carbonaria* Central versus South	0.25538	0.34157	0.00266
*T. hockingsi* North versus South	0.13197	0.16886	0.00251

*Note:* For the *Tetragonula hockingsi* North versus South subpopulation comparison, eight samples with evidence of admixture are excluded.

*T. hockingsi* , in contrast, clustered broadly into two populations (one comprising all samples from Southern and Central Queensland and another comprising those from Far North Queensland; Figure 2e). Admixture analysis indicated that the two most parsimonious population structures for *T. hockingsi* were either these same two populations, with limited hybridisation between them, or a single population comprising all individuals (Figure 2f; with CV errors very similar for these two scenarios).

Based on absolute nucleotide divergence (*dxy*), and assuming nuclear genome mutation rates in *Tetragonula* equivalent to those observed in honey bees and bumble bees (Yang et al. 2015; Liu et al. 2017), we estimate that *T. carbonaria* and *T. hockingsi* diverged 0.63–0.67 million years ago (one generation per year; Table S3). Estimated divergence times were in the range 0.36–0.53 Ma for the three *T. carbonaria* populations, and 0.34–0.37 Ma for the northern and southern *T. hockingsi* populations. Based on patterns of Tajima's D and f(ROH), the southern *T. hockingsi* population appears to have experienced historical population contractions and/or inbreeding (Supporting Information File 1).

**FIGURE 3:**
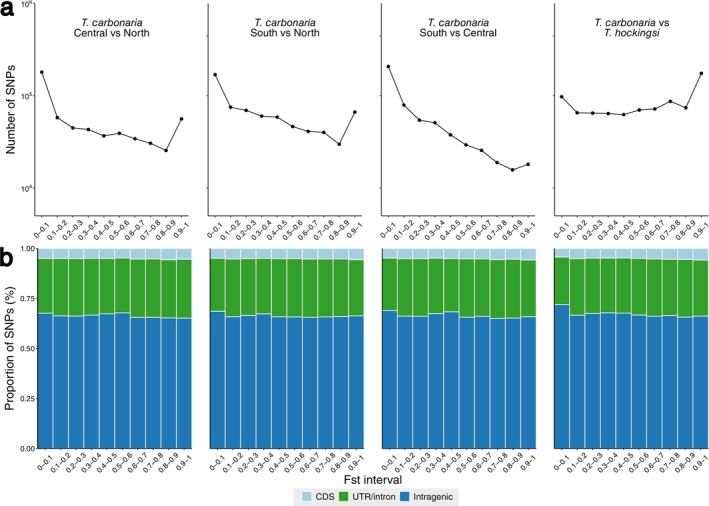
Genetic differentiation between *Tetragonula carbonaria* and *T. hockingsi*, and between *T. carbonaria* 's three discrete populations (North, Central and South) (a) Number of SNPs falling into different *F*_st_ intervals in each between‐population or between species comparison. (b) Proportion of SNPs falling into coding regions (CDS; light blue), untranslated (UTR)/intronic (green) and intragenic regions (blue) for each range of *F*_st_ values for each comparison.

Patterns of *F*_ST_ values between species, and between populations within a species, suggested that this genomic differentiation was partly driven by selection. Between *T. carbonaria* and *T. hockingsi* , the number of SNPs occupying different *F*_ST_ windows was broadly constant, except for increases at the lowest (*F*_ST_ = 0–0.1) and highest (0.9–1) bands (Figure 3a). Between the discrete *T. carbonaria* populations, SNP number generally decreased steadily when moving from lower to higher *F*_ST_ windows, but with an up‐tick of loci in the highest *F*_ST_ band (Figure 3a). For all comparisons, while the proportion of SNPs located within coding sequences varied across only a small range of values (e.g., between‐species: 4.36% and 5.82% in the lowest and highest *F*_ST_ windows respectively), there was nevertheless a strong and significant correlation between this proportion and *F*_ST_ (between‐species: Pearson's *ρ* = 0.873; *t* = 5.07; *p* < 0.001; within‐species: Pearson's *ρ* = 0.683–0.857; *t* = 2.65–4.71; *p* = 0.029–0.0015; Figure 3b). This is consistent with higher‐*F*_ST_ SNPs being disproportionately located in the genomic regions most exposed to selection. Among those genes that were most highly differentiated between species or populations (defined as genes with ≥ 3 CDS‐located SNPs with *F*_ST_ > 0.90; *T. carbonaria* vs. *T. hockingsi* : *n* = 370 genes, *T. carbonaria* Central vs. North: *n* = 44, Central vs. South: *n* = 24, and North vs. South: *n* = 56), four unique Biological Process GO terms were enriched in at least one comparison (Table S4). Notably, *Sensory Perception of Smell* was enriched in all comparisons, except that between the Central and Northern *T. carbonaria* populations.

### 3.3 Transcriptomic Analysis of Caste‐Biased Genes in *T. carbonaria*

#### 3.3.1 Patterns of Gene Expression in *T. carbonaria*

**FIGURE 4:**
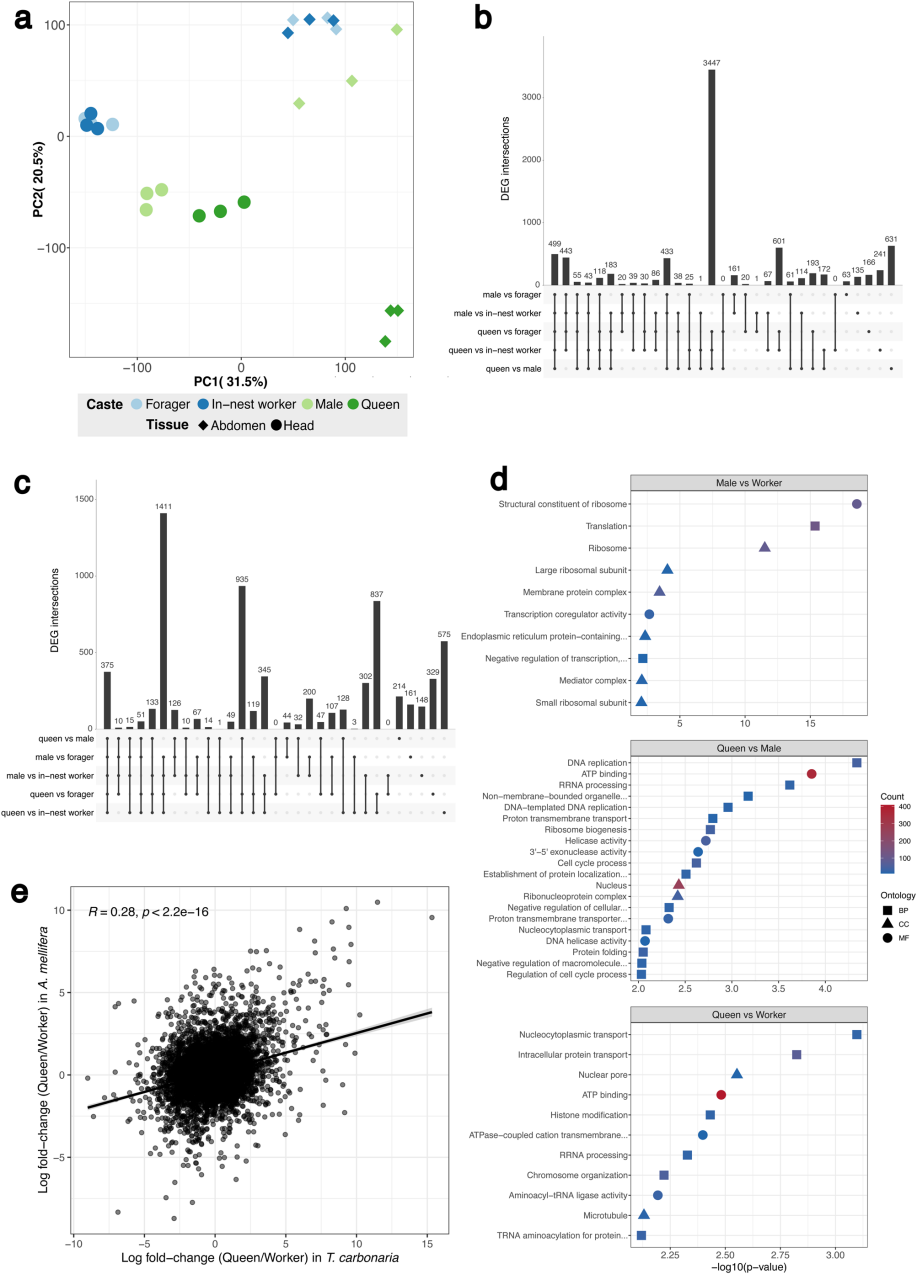
Patterns of caste‐biased gene expression in *Tetragonula carbonaria* (a) Principal component analysis of log‐transformed gene expression for 14,553 genes among queens, males, foragers and within‐nest workers (*n* = 6 for each group). (b) upSet plot showing numbers of differentially expressed genes shared between different pairs of castes within abdominal tissue. (c) upSet plot showing numbers of differentially expressed genes shared between different pairs of castes within head tissue. (d) GO terms enriched (unadjusted *p* < 0.01) among differentially expressed genes between workers, queens and males. Only the top 15 terms per comparison are shown. (e) Log(2) fold change in expression between queens and workers in *T. carbonaria* (*x*‐axis) and *Apis mellifera* (*y*‐axis; values obtained from Warner et al. 2019) for 8982 orthologous genes. Line of best fit generated in R via lm(); statistics via Pearson correlation.

Following filtering of genes with low expression, 13,892 of 24,389 genes (56.9%) remained for differential expression analysis. A majority (53.1%) of variation among tissues and castes was explained by the first and second principal components of variation, with tissue types separated primarily along PC1 (32.1% of variation), while castes within a given tissue type separated along both PCs (Figure 4a).

Overall, the largest transcriptomic divisions in head tissue were between sexuals (queens and males) and non‐sexuals (workers), whereas in abdominal tissue the largest divisions were between queens and non‐queens (males and workers); Figure 4a–c. For example, in abdomens, 3452 DEGs were ‘queen‐differentiating’ (shared across all comparisons between queens and other castes but not in any other pairwise comparison) compared to just 432 DEGs that were ‘sex‐differentiating’ (shared across all comparisons between males and females but not in any comparison between females; Figure 4b).

Because very little gene expression differentiation was detected between foragers and within‐nest workers in either tissue (28 and 14 DEGs in heads and abdomens respectively; Figure 4b,c), we consolidated these samples into a single ‘worker’ category for further caste comparisons. Combining caste and tissue into a single DESeq2 model and then applying pairwise contrasts, we identified 5581 total DEGs between queens and workers (2397 up in queens, 3184 up in workers); 4067 between queens and males (1640 up in queens, 2427 up in workers); and 2689 between males and workers (1292 up in males, 1397 up in workers), with a range of distinct GO terms associated with the DEGs of each caste comparison; Figure 4d; Tables S5–S7.

Taking only genes with a reciprocal best BLAST hit between *T. carbonaria* and the honey bee *Apis mellifera* (*n* = 8982; honey bee data taken from Warner et al. 2019), there was a strongly significant correlation in log fold change (LFC) of expression with caste (queen/worker) between the species (Pearson's *R* = 0.28; *p* < 0.0001; Figure 4e; Table S8). However, the sets of DEGs defined as caste‐biased in the two species did not overlap significantly beyond chance, either for worker‐biased genes (381 DEGs shared from gene sets of 1065 and 3469 for *T. carbonaria* and *A. mellifera* respectively; one‐sided hypergeometric test, *p* = 0.98) or queen‐biased genes (191 DEGs shared from gene sets of 688 and 4561; one‐sided hypergeometric test, *p* = 1). Focusing on specific genes known to play a role in *A. mellifera* caste expression, we found that vitellogenin (*Vg*) exhibited strongly concordant expression between the species, being strongly queen biased in both. However, juvenile hormone esterase precursor (*Jhe‐pre*) and insulin‐like peptide receptor (*InR*), both of which are strongly worker‐biased in *A. mellifera* , showed only weak worker bias in *T. carbonaria* . Juvenile hormone acid methyltransferase (*Jhamt*) showed the opposite pattern, being much more strongly worker‐biased in *T. carbonaria* than *A. mellifera*. Thus, caste‐biased gene expression differences, though broadly conserved in their directionality across these two independently superorganismal bee lineages, appear to be only weakly conserved in terms of the strength of their differential expression.

#### 3.3.2 Tests of the Relaxed Constraint and Adapted Worker Hypotheses

**FIGURE 5:**
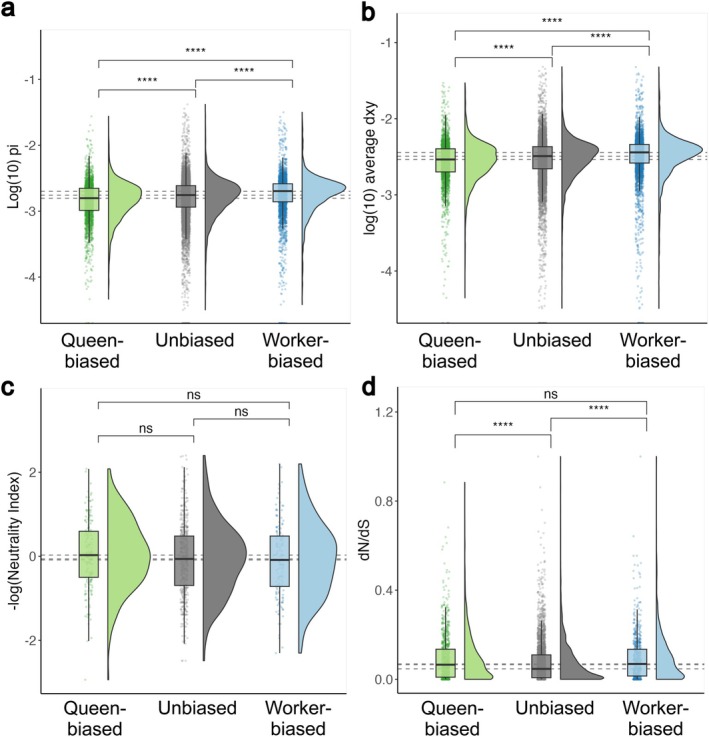
Genomic parameters of *Tetragonula carbonaria* genes grouped by queen‐worker caste expression bias. (a) Log(10) nucleotide diversity; (b) population sequence divergence (*dxy*) between *T. carbonaria* and *T. hockingsi* ; (c) −log2 neutrality index (NI); and (d) dN/dS ratio. Significance values indicated by four asterisks (*p* < 0.001) or ns (non‐significant) are the result of two‐sided Wilcoxon tests following correction for multiple comparisons, and dashed lines represent median values of the three sets of genes.

We tested whether caste‐biased DEGs among females (queens vs. workers) exhibited significant differences in their genomic signatures. In line with the expectations of the Relaxed Constraint hypothesis, worker‐biased genes exhibited greater nucleotide diversity than either queen‐biased or caste‐unbiased genes (Wilcoxon rank sum test FDR‐adjusted *p* = 0.002 in the comparison between queen‐biased and caste‐unbiased genes; *p* < 0.0001 in both other contrasts; Figure 5a; Table S9). Worker‐biased genes also exhibited greater absolute sequence divergence between *T. carbonaria* and *T. hockingsi* (*dxy*) than did either queen‐biased or caste‐unbiased genes (Wilcoxon rank sum *p* < 0.0001 in all contrasts; Figure 5b; Table S9). The latter result is predicted under a model of relaxed constraint, but might also be consistent with the Adapted Worker hypotheses, since both relaxed selection and directional selection could increase sequence divergence between two lineages. However, relative genomic differentiation (*F*_ST_) exhibited the opposite pattern, being higher than average in queen‐biased genes and lower than average in worker‐biased genes (Wilcoxon rank sum *p* ≤ 0.01 in all contrasts; Figure S1). This combination of elevated *F*_ST_ and decreased *dxy* is indicative of recurrent bouts of selection that occur both before and after a phylogenetic split (Cruickshank and Hahn 2014; Irwin et al. 2016; Jiang et al. 2023), and its occurrence in queen‐biased genes (and opposite in worker‐biased genes) therefore supports a model of relatively relaxed selection on worker genes.

Other tests of selection acting on caste‐biased genes did not provide support for either of our two hypotheses. Caste‐biased genes did not exhibit biases in Direction of Selection (DoS) nor Neutrality Index (NI) coefficients compared to queen‐biased and caste‐unbiased genes (Wilcoxon rank sum *p* > 0.16 in all contrasts; Figures 4c and S2; Table S9). Moreover, dN/dS, another measure of the direction and strength of selection, was slightly higher for caste‐biased genes than for unbiased genes (Wilcoxon rank sum *p* < 0.0001 in both contrasts; Figure 5d; Table S9) but did not differ between those that were worker‐biased and those that were queen‐biased (Wilcoxon rank sum *p* = 0.93; Figure 5d; Table S9). Given that a previous study of the honey bee *A. mellifera* found strong support for elevated positive selection on worker genes (Harpur et al. 2014), we re‐analysed those data to confirm that the contrasting results between *Apis* and *Tetragonula* were not due to methodological differences in the analysis approach. We took allele frequency data from Harpur et al. (2014), downsampled those data to match the sampling depth obtained in the present study, calculated NI and DoS for these data using the same methods as above, and then took the subset of data including only those genes with reciprocal best BLAST hits between *A. mellifera* and *T. carbonaria* . We found that within this set of orthologues, worker‐biased genes obtained independently by Warner et al. (2019) in *A. mellifera* still showed elevated NI and DoS compared to queen‐biased or caste‐unbiased genes, while those in *T. carbonaria* did not (Figure S3).

If, as predicted by the Adapted Worker hypothesis, worker genes disproportionately code for evolutionarily novel functions, such genes might also be disproportionately likely to be evolutionarily novel. In support of this possibility, worker‐biased genes in *T. carbonaria* exhibited much shorter average effective lengths than caste‐unbiased genes, which were likewise much shorter than queen‐biased genes (Wilcoxon rank sum *p* < 0.0001 in all contrasts; Figure S4; Table S9); this is the expected pattern given that there is a positive correlation between evolutionary age and gene length (Grishkevich and Yanai 2014; Heyn et al. 2015). As a more direct test of evolutionary novelty, we also assessed the phylostratum to which each gene belonged. Evolutionarily ancient genes showed a strong association with caste bias, with queen genes being much more likely, and worker genes much less likely, than caste‐unbiased genes to have a common ancestor in Opisthokonta or earlier (66.9%, 42.3% and 50.2% of queen‐biased, worker‐biased, and caste‐unbiased genes respectively; Fisher's exact test *p* < 0.0001; Table S9). The most evolutionarily novel stratum of genes (those with a common ancestor more recent than Anthophila) also showed an association with caste, being less frequent among queen‐biased genes than among worker‐biased or caste‐unbiased genes (12.1%, 20.4% and 22.5% of queen‐biased, worker‐biased, and caste‐unbiased genes respectively; Fisher's exact test *p* < 0.0001; Figure S5). However, worker genes were slightly less likely than caste‐unbiased genes to be found in this most recent phylostratum (Fisher's exact test *p* = 0.008).

We also tested for differences among caste‐biased genes in terms of GC content, a measure that does not directly relate to either the Relaxed Constraint or Adapted Worker hypotheses but which correlates strongly with both caste bias and nucleotide diversity in *A. mellifera* , with queen genes being AT‐biased and worker genes being GC‐biased (Kent et al. 2012). We found the same effect in *T. carbonaria* : caste‐unbiased genes were slightly AT‐biased, queen‐biased genes were strongly AT‐biased, and worker‐biased genes were slightly GC‐biased (Wilcoxon rank sum *p* < 0.0001 in all contrasts; Figure S6; Table S9). Similarly to *A. mellifera* (Beye et al. 2006; Honeybee Genome Sequencing Consortium 2006; Kent et al. 2012), in *T. carbonaria* we found a significant positive correlation between the GC content of genes and nucleotide diversity, both within and across sets of caste‐biased genes (Spearman's rho = 0.21, *p* < 0.0001 across all genes).

Finally, because our population genomic analyses (above) indicated that the sampled *T. carbonaria* and *T. hockingsi* constituted multiple distinct populations, we wished to account for the possibility that the combination of multiple populations within a single analysis might have skewed our results. We therefore re‐ran each sociogenomic comparison using only the southern populations of *T. carbonaria* and *T. hockingsi* , as identified by our population genomic analysis. We found the patterns of sociogenomic results in each case were qualitatively the same when only southern populations were included (Table S10).

## 4 Discussion

### 4.1 Genomic Signatures of Sociality in *T. carbonaria*

The phenotypically distinct castes of *Tetragonula* arise from the same genome (Nunes et al. 2015) and thus must arise from differences in regulation (e.g., gene expression) between the castes, as is the case for other social hymenopterans (Okwaro and Korb 2023; Orr and Goodisman 2023). We found strong caste‐biased and sex‐biased patterns of gene expression in both the heads and abdomens of *T. carbonaria* , with several 100 genes being differentially had between queens, workers and males. In particular, the abdomens of queens exhibited a distinct transcriptomic signature, consistent with a high volume of transcriptomically active ovarian tissue (Bueno et al. 2020). The common ancestor of stingless bees and honey bees likely exhibited worker and queen castes but not obligate worker sterility (Cardinal and Danforth 2011). We found the transcriptomic signatures of caste differentiation to be only weakly conserved between these two independently superorganismal bee taxa: while caste‐related fold changes were correlated overall between *T. carbonaria* and *A. mellifera* , only a small subset of the ~2000 significantly caste‐biased genes in *T. carbonaria* showed overlap with those in *A. mellifera* . These results are in line with comparative studies of more distantly related social insect taxa, which have found that social insect castes may be differentiated by a small number of ‘core’ genes or pathways across different taxa, but that the majority of genes involved in caste differentiation are lineage‐specific (Toth et al. 2010; Berens et al. 2015; Dogantzis et al. 2018; Taylor et al. 2024; Wyatt et al. 2023).

The Relaxed Constraint hypothesis predicts that, because genes principally expressed in workers (and thus assumed to principally encode worker traits) impact inclusive fitness only indirectly, they will experience weaker selective constraint and show signatures of relaxed selection (Linksvayer and Wade 2009). The Adapted Worker Hypothesis (Harpur et al. 2014) predicts that worker‐biased genes are more likely to exhibit signatures of recent positive selection as evolutionarily novel phenotypes associated with sociality are primarily expressed by workers. In principle, these hypotheses are not mutually exclusive because the effect of relaxed selection will manifest mostly as an increase in low frequencies of mildly deleterious alleles, while those alleles that are strongly favourable are still positively selected for and become fixed (despite positive selection being less efficient, all else being equal, than it would be for traits under direct selection). Indeed, a key effect of the heightened genetic polymorphism caused by relaxed selection may be that there is more opportunity for novel functions to arise, both within and between populations (True and Carroll 2002); that is, indirect selection may help set the stage for the novel and remarkable adaptations of the worker caste (Linksvayer and Wade 2009). In practice, however, it is challenging to identify signals of *both* relaxed selection and enhanced positive selection when averaging across large sets of differentially expressed genes, and previous studies in other social insects have generally concluded one or the other trend to be dominant. In *T. carbonaria*, we find both higher nucleotide polymorphism within populations and higher absolute sequence divergence between populations for worker‐biased genes, relative to both queen‐biased and unbiased genes. Intriguingly, absolute and relative divergence exhibit opposite patterns in caste‐biased genes, with queen genes having elevated *F*_ST_ but decreased *dxy* relative to the genomic background, and worker genes showing the inverse pattern. The concurrence of high *F*_ST_ and low *dxy* is considered indicative of recurrent selection occurring both before and after a population split (Cruickshank and Hahn 2014; Irwin et al. 2016; Jiang et al. 2023). The presence of this signature in queen genes and its obverse in worker genes thus supports the notion that queen genes have experienced strengthened selection, and worker genes relaxed selection, in *T. carbonaria* both before and after its split with *T. hockingsi* . Since this common ancestor is presumed to have possessed worker sterility, this pattern thus strongly supports the relaxed selection hypothesis but not the adapted worker hypothesis.

Despite the nucleotide diversity and population divergence statistics discussed above, we did not find differences between sets of caste‐biased genes when analysing two closely related measures that evaluate selection by measuring the proportion of synonymous and nonsynonymous sites that are fixed vs. polymorphic between species: Neutrality Index (NI) and Direction of Selection (DoS). In *Apis mellifera* , equivalent analyses have shown worker genes to be significantly enriched for signatures of positive selection, suggesting that worker adaptation has been a key component of social evolution in that lineage (analyses based on comparisons between *A. mellifera* and *A. cerana* ; Harpur et al. 2014). As we were able to rule out the influence of most methodological differences (such as those used to detect DEGs or to calculate signatures of selection), the absence of an equivalent signature in *T. carbonaria* could reflect genuine differences in the way that selection acts on caste‐biased genes in these two social bee clades. *Apis* and *Tetragonula* do vary in ways that might impact the efficacy of selection acting on worker‐biased genes. In particular, *A. mellifera* queens are extremely polyandrous (~11–17 mates/queen; Tarpy et al. 2013), while *T. carbonaria* queens are strictly monandrous (Green and Oldroyd 2002). By increasing genetic variation among workers, however, polyandry reduces the correlation between any individual worker's genotype and the overall colony phenotype (Calderone and Page 1992), and should thereby further reduce the strength of selection (i.e., efficacy of positive selection) on worker‐expressed genes (Linksvayer and Wade 2009). That is, differences in mating system in this case should increase, rather than decrease, the likelihood of finding elevated positive selection in *T. carbonaria* genomes relative to *Apis*. Alternatively, it may be that the amount of differentiation between workers of *A. mellifera* and *A. cerana* is greater than that present between *T. carbonaria* and *T. hockingsi* . Workers of the two *Apis* species have well‐documented lineage‐specific differences, including in their nest defence, foraging and thermoregulatory behaviours (Tan et al. 2012; McClenaghan et al. 2019), and their size (Ruttner 2013). By contrast, workers of *T. carbonaria* and *T. hockingsi* have comparatively similar phenotypes (Dollin et al. 1997; Brito et al. 2014), and as a result may have experienced less divergent selection than that which has occurred among *Apis* species. The two *Tetragonula* species are also only recently diverged, which may decrease the chances of detecting significant differences in direct signatures of selection: we estimate that *T. carbonaria* and *T. hockingsi* split approximately 0.65 Ma, an order of magnitude more recently than the split between *A. mellifera* and *A. cerana* (~7.5 Ma; Dogantzis et al. 2018). Indeed, we found that for the majority of genes, not enough SNPs were present between the two *Tetragonula* species to allow NI or DoS to be accurately calculated (Table S10), and our analyses of these two metrics may therefore be less robust than those using other metrics.

While the overall ratio of nonsynonymous substitution (dN/dS) in *T. carbonaria* did not differ between worker‐biased and queen‐biased genes, it was higher in both sets relative to caste‐unbiased genes. This same result has been found for dN/dS in caste‐biased genes in honey bees and in the superorganismal ants *Monomorium pharaonis* (Warner et al. 2019) and *Lasius niger* (Lucas et al. 2017). More generally, higher rates of molecular evolution have been observed in many cases of morph‐biased genes, where morphs may be castes, sexes or other phenotypic variants within a species (e.g., Whittle and Johannesson 2013; Helanterä and Uller 2014; Purandare et al. 2014; Wang et al. 2015). This effect may reflect the fact that loosely regulated genes (those under relaxed selection) are more likely to become caste‐biased, rather than genes becoming subject to relaxed selection as a result of their caste bias (Helanterä and Uller 2014; Lucas et al. 2017; Mikhailova et al. 2024). In *T. carbonaria* , therefore, one possibility is that both worker‐ and queen‐biased genes were originally co‐opted for roles in caste expression due to weak regulation (producing the observed pattern in dN/dS), but that queen‐biased genes were subsequently subject to stronger regulation due to their role in the reproductive phenotype, while worker genes have remained loosely regulated (producing the observed pattern of higher nucleotide diversity and between‐species sequence divergence in worker genes).

Evidence from multiple social insect taxa suggests that worker‐biased genes have arisen more recently on average than queen‐biased genes (Behl et al. 2018; Warner et al. 2019). In *Tetragonula*, we find that worker genes are significantly shorter (and queen genes significantly longer) than caste‐unbiased genes, a measure that is known to correlate with evolutionary age, and worker genes were less likely (and queen genes more likely) to have ancient origins than caste‐unbiased genes. However, we did not find that worker genes were, on average, disproportionately likely to belong to the most taxonomically restricted phylostratum, which should have been the case if such genes had mostly arisen secondarily to the evolutionary origins of sterile (or mostly sterile) workers. Instead, our results support the notion that the genetic ‘groundplan’ for sociality pre‐dates the evolution of an obligately sterile worker caste.

### 4.2 Population Differentiation Within and Between *Tetragonula* Species

Phylogenetic studies regularly turn up multiple discrete lineages within what was previously considered a single species (Bickford et al. 2007), and it is increasingly clear that cryptic species represent a significant portion of global biodiversity (Jörger and Schrödl 2013; Struck et al. 2018; Benefits sharing statement; Termignoni‐Garcia et al. 2022). Even where taxa are best considered a single species, they may have significant population structure across their range that is not readily apparent from distribution data alone. An understanding of population structure is therefore an important foundation for studies of genome evolution, particularly for species with cryptic morphologies. We identified significant within‐species structure in both *T. carbonaria* and *T. hockingsi* , consistent with that suggested by previous studies of mitochondrial DNA and microsatellites (Franck et al. 2004; Brito et al. 2014; Françoso et al. 2019; Françoso et al. 2023) and reduced representation sequencing (Nacko 2023; Law et al. 2024). *T. carbonaria* comprises at least three genetically distinct populations that are likely allopatric (northern Queensland, central Queensland and southern Queensland to Sydney), while *T. hockingsi* comprises at least two populations (northern Queensland and central/southern Queensland), with some admixture between them. Population structure in both species is likely the product of cycles of climate change that occurred in the Australian Wet Tropics throughout the Pleistocene (Byrne 2008; Hawlitschek et al. 2012), and which have similarly shaped the diversity and distribution of many forest taxa on the continent's north‐eastern coast. Indeed, our estimation of divergence times places all species and population divisions for *T. carbonaria* and *T. hockingsi* in the Middle Pleistocene, 0.35–0.75 Ma, which is similar to a previous estimate for species divergence of 0.5 Ma (based on a dated phylogeny of the nuclear gene Ef1‐alpha; Françoso et al. 2019).

We found some evidence that overall differences between these two species were selectively driven: there was a significant, though small, increase in the proportion of SNPs found in coding sequences at higher values of *F*_ST_ (Figure 3). Among the genes with particularly strong signatures of selectively driven differentiation between the species, and between populations within a species, were those enriched for olfactory functions, which may reflect differences in a range of behaviours from preferred diet or nesting materials to mate recognition (Conchou et al. 2019). For example, while *T. hockingsi* males have recently been shown to join mating aggregations of *T. carbonaria* in southern Brisbane where the species co‐occur, they do not attempt to mate with *T. carbonaria* virgin queens at short range (Paul et al. 2023). This suggests that olfactory mate recognition cues likely play a role in preventing interspecific hybridisation (Hereward et al. 2020; Paul et al. 2023). Whether such pre‐zygotic barriers also occur between the three *T. carbonaria* populations remains to be investigated. The extent and fitness of interpopulation hybrids in this species is an important consideration for their commercial use, as hives are increasingly being translocated between regions. More generally, *Tetragonula* stingless bees are a promising system not only for the provision of insights into the genomics of social evolution, but also the genomics of population differentiation and speciation.

## Author Contributions

B.A.H., R.G., B.P.O. and B.A.T. conceived and designed the study. B.A.T. performed the gene expression and population genomic analyses with input from B.A.H. and R.G. R.G. and J.D. collected the samples. G.B., B.A.T., B.A.H. and G.P.S. performed molecular lab work. E.S. coordinated genome assembly and annotation. B.A.T. and R.G. wrote the main manuscript text with input from all authors.

## Benefits sharing statement

Benefits sharing statement: This research was made possible by extensive collaboration between Australian, European and North American co‐authors and collaborators, in line with our group's commitment to international scientific partnerships. Among other topics, this research addresses a priority concern, namely the potential anthropogenic breakdown of natural population barriers in an ecologically important clade of pollinators. Benefits from this research include the sharing of our data, methods and results on public databases.

## Conflicts of Interest

The authors declare no conflicts of interest.

## Supporting information


Data S1.

Data S2.

Data S3.

## Data Availability

Genome assembly files are available under NCBI GenBank reference GCA_032399595. Population genomic and RNA‐seq data are available under NCBI BioProject Reference PRJNA1014002.
