# Supplementary material for: Multi‐Omic Analysis Reveals Population Differentiation and Signatures of Social Evolution in Tetragonula Stingless Bees
Source: Mol Ecol. 2025 Jun 11;34(13):e17823. doi: 10.1111/mec.17823 (PMC12186725; doi:10.1111/mec.17823)
Supplement: Supplementary file 1 — Data S1. [file MEC-34-e17823-s001.pdf]

Supplemental Information File 1

Multi-omic analysis reveals population differentiation and signatures of social evolution in *Tetragonula* stingless bees

Benjamin A. Taylor, Garett P. Slater, Eckart Stolle, James Dorey, Gabriele Buchmann, Benjamin P. Oldroyd, Rosalyn Gloag*, Brock A. Harpur*

***Tetragonula carbonaria* genome assembly and annotation**

We performed an initial genome assembly using one *T. carbonaria* male (haploid) pupa from a colony sourced from the University of Sydney apiary, Sydney, New South Wales in 2017. We removed the pupa's head and extracted DNA from the remaining tissue according to the 10x Genomics HMW gDNA Extraction from Single Insects protocol (2018: Manual No. CG000145). The DNA was then used to construct a sequencing library with 10X Genomics technology at the Australian Genome Research Facility (AGRF). We later collected further males from a mating aggregation at the University of Sydney (2018) onto dry ice and delivered them to Dovetail Genomics (www.dovetailgenomics.com) for extraction and sequencing of pure DNA and endogenous chromatin from a single male using a proximity ligation-optimized protocol (Chicago and Dovetail Hi-C). To create the 10x Chromium assembly we used Supernova 2.0.1 (Visendi 2022) on the 10x Chromium linked-read data using default parameters. To remove heterozygous, duplicated, scaffolds we used purge-haplotigs v1.04 (Roach et al. 2018) with *-l 4 -m 80 -h 90*. We used RepeatMasker v4.07 (Smit et al. 2013) to identify and soft-mask repeat regions of the genome.

To further refine this assembly, we generated paired short-read (Illumina) and long-read data (Nanopore) from DNA extracted separately from two black eyed female pupae sampled from Brisbane, Queensland, Australia. We extracted the DNA of the first of the two female pupae via phenol-chloroform extraction with ethanol precipitation, followed by library preparation and 150 bp paired-end sequencing on a HiSeq4000. We filtered short read sequences of optical duplicates using Clumpify v37.50 (optical distance 5000, k=31; Bushnell 2014); quality and adapter trimming were performed using Fastp v0.19.4 (Chen et al. 2018) with default settings and a minimum read length of 100 bp. From the second female pupal sample, high molecular weight DNA was extracted via proteinase K/RNAse A digestion in Tris Buffer, followed by purification with AMPur magnetic beads. This DNA was sequenced on one MinION R10.0 flowcell for 48h using a GridION device. We performed basecalling on GridION with Albacore. We trimmed adapters from long reads using Porechop v0.2.4 (Wick et al. 2017). Following extraction and filtering of sequencing data, we constructed three additional genome assemblies. One assembly was generated using combined short- and long-read data with MaSuRCA v3.4.1 (Zimin et al. 2013) and Flye v2.5 (Kolgomorov 2019). A second assembly was generated using Canu v1.8 (Koren et al. 2017) followed by two rounds of polishing using the short reads with pilon v1.22 (Walker et al. 2014), and a third using wtdbg2 v2.5 (Ruan & Li 2020) with default settings and long reads only.

We finally merged these three assemblies together with our original 10X assembly using CSA v2.6 (Kuhl et al. 2020), followed by 2 rounds of polishing with pilon v1.22 (Walker et al. 2014) and cleaning with purge_dups v1.0.1 (Guan et al 2020). We soft-masked repeats using

RepeatMasker v4.07 (Smit et al. 2013), bioawk (Quinlan & Li 2015) and seqtk (Li 2013) using a repeat library generated from 0.25X short reads and dnaPipeTE (Goubert et al. 2015) retaining only high abundance repeats. We determined the completeness of the final assembly (*TetCarb_2.0*; GenBank GCA_032399595.1) using BUSCO v4.0.6 (Manni et al. 2021) with the hymenoptera_odb10 dataset.

For annotation, we generated transcriptome assemblies from our RNAseq data (NCBI Sequence Read Archive, SRR1503044) using Trinity v2.9.1 (Grabherr et al. 2011), stringtie v2.1.1 (Pertea et al. 2015) with hisat2 v2.2.1 (Kim et al. 2019) and BinPacker v1.0 (Liu et al. 2016). After trimming adapters and filtering for length and quality, 22.1M reads remained for transcriptome assembly (Trinity: 131.8k transcripts with an average size of 598bp; stringtie: 53.3k transcripts; binpacker: 52.9k transcripts, average size 727bp). The Trinity transcriptome assembly was used to train PASA v2.4.1 (Haas et al. 2003). Next, Funannotate used gene predictions from PASA, Genemark-ES (Ter-Hovhannisyan et al. 2008), Snap v2006-07-28 (Korf 2004), glimmerHmm v3.0.4 (Majoros et al. 2004), Augustus v.3.3.3 (Keller et al. 2011), and CodingQuarry v2.0 (Testa et al. 2015) together with protein alignments in EVidenceModeler v1.1.1 (Haas et al. 2008). We removed gene models that were too short, gap-spanning or repeat-overlapping and tRNA genes were detected with tRNAscan-SE v2.0.6 (Chan & Lowe 2019).

Genes were functionally annotated using Pfam v33.1 (Mistry et al 2020), the UniProt database v2018_11 (UniProt Consortium 2022; based on protein sequences from 11 bee species, **Table A1**), EggNog (eggnog_4.5/hmmdb databases: Arthropoda, Insecta, Hymenoptera, Drosophila; Huerta-Cepas et al. 2016), MEROPS v12.0 (Rawlings et al. 2017), CAZYmes in dbCAN v7.0 (Yin et al. 2012), BUSCO Hymenoptera models v3.0.2 with Hymenoptera odb9 (Simão et al. 2015), SignalP v4.1 (Petersen et al. 2011), and InterProScan5 v81.0 (Jones et al. 2014).

Table A1. 11 bee species with protein data used to functionally annotate the genome assembly of *Tetragonula carbonaria.*

**Species name**	**NCBI reference**
Bombus impatiens	GCF000188095.2
B. terrestris	GCF000214255.1
Apis mellifera	GCF003254395.2
Melipona quadrifasciata	GCA001276565.1
Eufriesea mexicana	GCF001483705.1
Frieseomelitta varia	GCA011392965.1
Megachile rotundata	GCF000220905.1
Habropoda laboriosa	GCF001263275.1
Dufourea novaeangliae	GCF001272555.1
Megalopta genalis	GCF011865705.1
Nomia melanderi	GCF003710045.1

Evidence of inbreeding as measured in terms of long runs of homozygosity

To test whether individual populations might have been subject to historical bottlenecks, we searched for signatures of past inbreeding in the form of long runs of homozygosity (ROHs). To calculate the proportion of the genome occupying ROHs, we first simulated a single fully homozygous sample and used PLINK v2.0 (Chang et al. 2015) to identify runs of homozygosity with the following settings: --homozyg-snp 50; --homozyg-density 60; --homozyg-gap 500; --homozyg-het 50000; --homozyg-window-snp 50; --homozyg-window-het 1; --homozyg-window-missing 5; --homozyg-window-threshold 0.05. Doing so allowed us to assess the theoretical maximum length of ROHs that could feasibly be identified in an individual sample. To test different theoretical maximum lengths of ROH, we identified ROHs repeatedly setting --homozyg-kb to 50, 100 or 500. We then identified actually existent ROHs in each individual sample using the same settings. f(ROH) for a given sample at a given minimum ROH length was calculated by dividing the observed sum of ROH lengths in that sample by the sum of ROH lengths in the simulated homozygous sample for the same settings. Population f(ROH) for a given ROH length was taken as the average f(ROH) for all samples in that population.

Evidence of inbreeding as measured in terms of long runs of homozygosity for each population were similar (**Table A2**), differing by less than an order of magnitude in most cases. The combined *T. hockingsi* population, and especially the putative Southern *T. hockingsi* subpopulation, both exhibited elevated values of fROH at lower (50 & 100kb) lengths of ROH, but this disparity was not evident at the longest (500kb) length of ROH measured (**Table A2**). The putative Southern *T. hockingsi* population also exhibited a high value of Tajima's D (Schmidt & Pool 2002), as did the combined set of *T. carbonaria* samples. In the latter case, this elevated value likely reflects the inclusion of multiple distinct populations within a single analysis (thereby simulating the effects of balancing selection). However, in the case of Southern *T. hockingsi*, together with elevated values of f(ROH), elevated Tajima's D may plausibly reflect historical population contractions and/or inbreeding.

Table A2. Population genomic statistics for each population of *Tetragonula* samples. For the *T. hockingsi* subpopulation comparison, eight samples with evidence of admixture are excluded.

Species	Population	Number of samples	SNPs	LD-filtered SNPs	fROH(50)	fROH(100)	fROH(500)	Tajima's D
All samples + outgroups	Combined	72	838406	153859				
T. carbonaria	Combined	40	689431	250595	0.066	0.045	0.014	2.263
	Northern	10	641175	72036	0.047	0.035	0.006	0.889
	Central	6	472612	22605	0.094	0.064	0.021	0.691
	Southern	24	505616	240418	0.046	0.035	0.015	0.275
T. hockingsi	Combined	29	821109	166937	0.212	0.112	0.024	0.270
	Northern	7	816507	69839	0.094	0.044	0.005	0.452
	Southern	14	813810	26205	0.282	0.145	0.024	1.771
