# Supplementary material for: Multi‐Omic Analysis Reveals Population Differentiation and Signatures of Social Evolution in Tetragonula Stingless Bees
Source: Mol Ecol. 2025 Jun 11;34(13):e17823. doi: 10.1111/mec.17823 (PMC12186725; doi:10.1111/mec.17823)
Supplement: Supplementary file 2 — Data S2. [file MEC-34-e17823-s003.pdf]

Supplemental Figures

Multi-omic analysis reveals population differentiation and signatures of social evolution in *Tetragonula* stingless bees

Benjamin A. Taylor, Garett P. Slater, Eckart Stolle, James Dorey, Gabriele Buchmann, Benjamin P. Oldroyd, Rosalyn Gloag*, Brock A. Harpur*

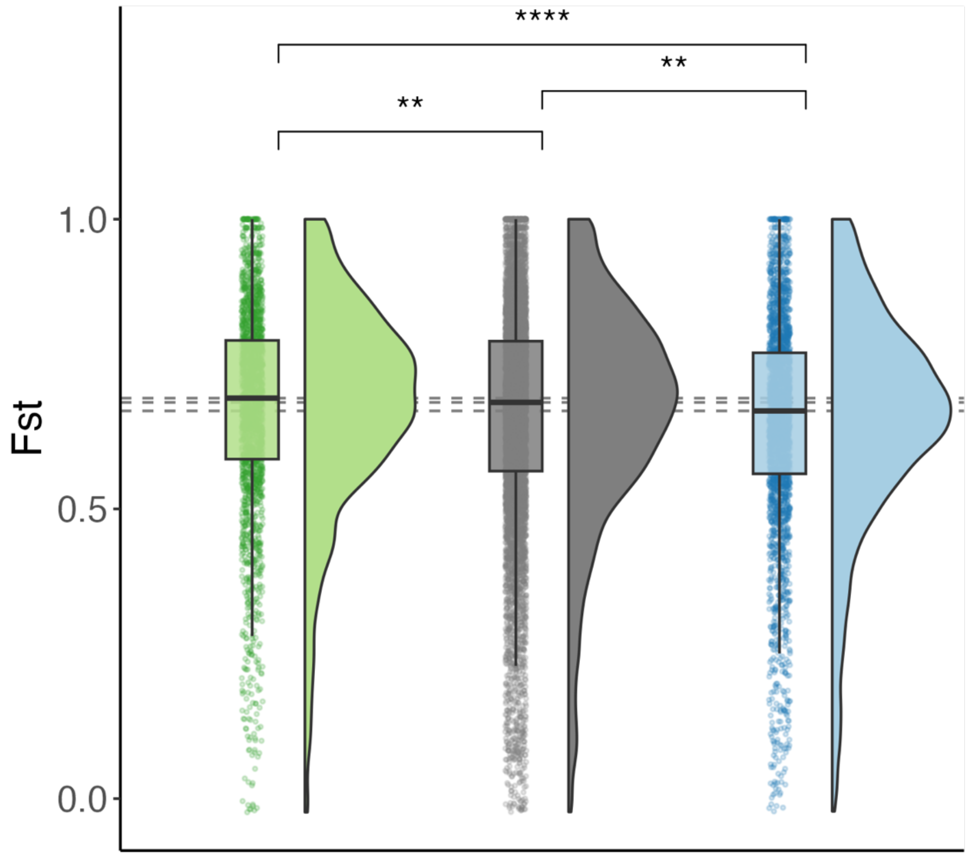

Figure S1. Relative population differentiation (Fst) between *Tetragonula carbonaria* and *T. hockingsi* of *T. carbonaria* genes, grouped by caste-biased gene expression (left, green: queen-biased; middle, grey: caste-unbiased; right, blue: worker-biased). Significance values indicated by asterisks are the result of two-sided Wilcoxon tests following correction for multiple comparisons, and dashed lines represent median values of the three sets of genes.

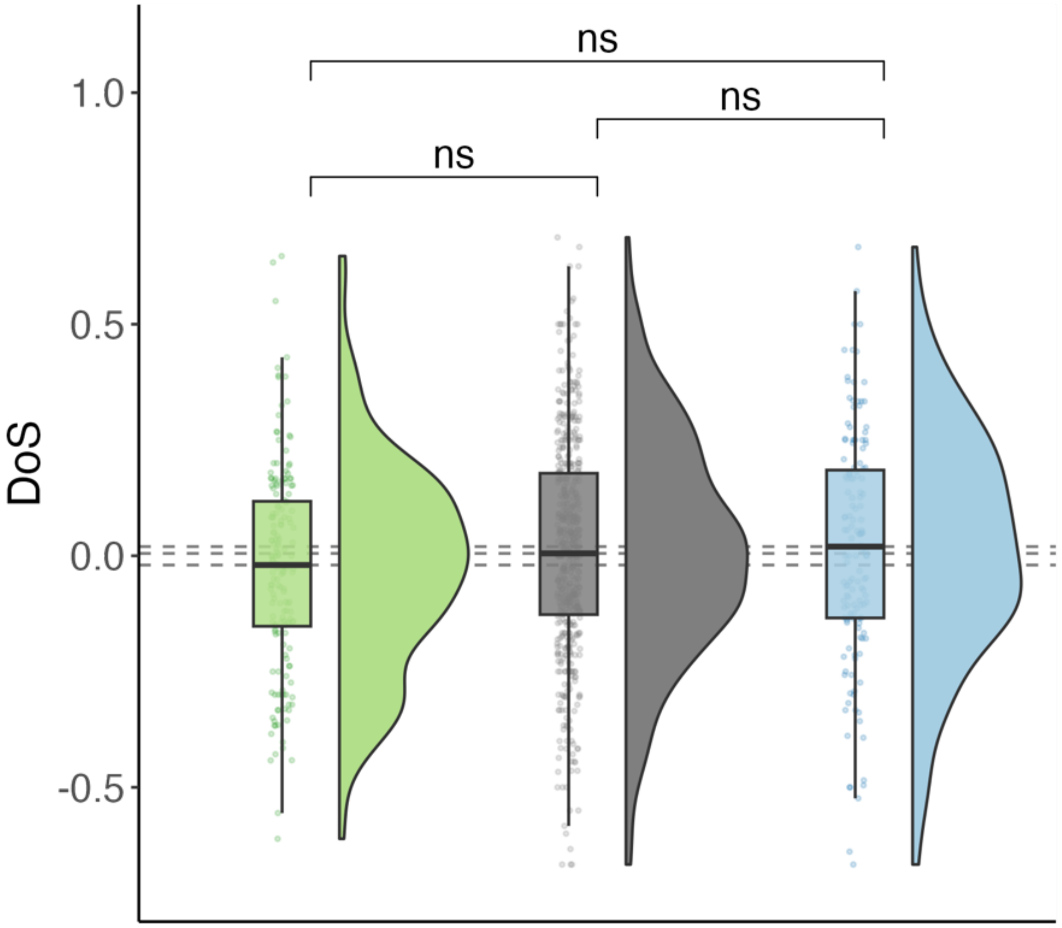

Figure S2. Direction of selection coefficient (DoS) of *T. carbonaria* genes, grouped by caste-biased gene expression (left, green: queen-biased; middle, grey: caste-unbiased; right, blue: worker-biased). Significance values indicated by asterisks are the result of two-sided Wilcoxon tests following correction for multiple comparisons, and dashed lines represent median values of the three sets of genes.

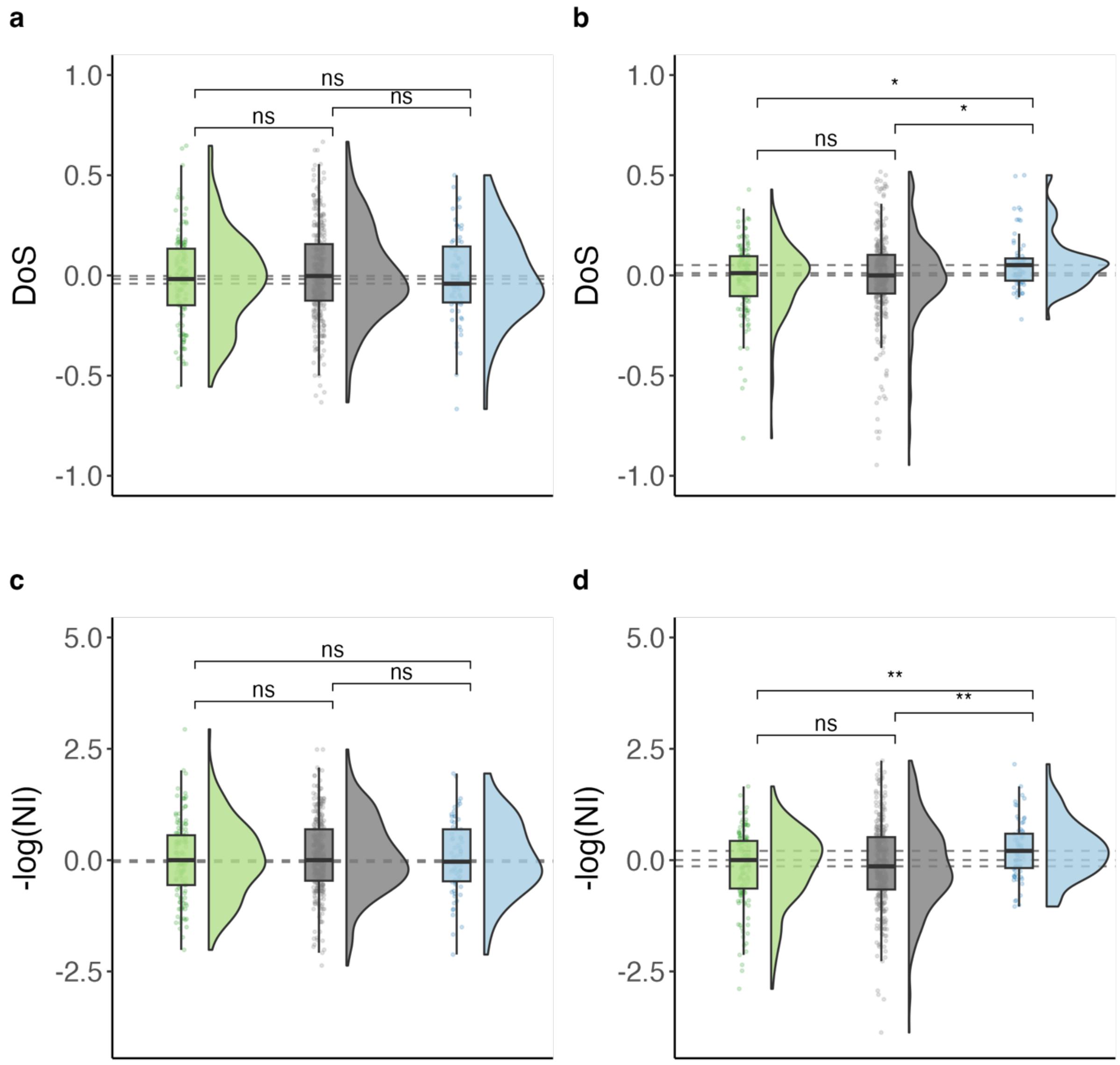

Figure S3. Direction of selection coefficient (DoS) and negative log neutrality index (NI) of reciprocal BLAST best hits between *T. carbonaria* (**a** and **c**) and *Apis mellifera* (**b** and **d**), grouped by caste-biased gene expression (left, green: queen-biased; middle, grey: caste-unbiased; right, blue: worker-biased). Significance values indicated by asterisks are the result of two-sided Wilcoxon tests following correction for multiple comparisons, and dashed lines represent median values of the three sets of genes.

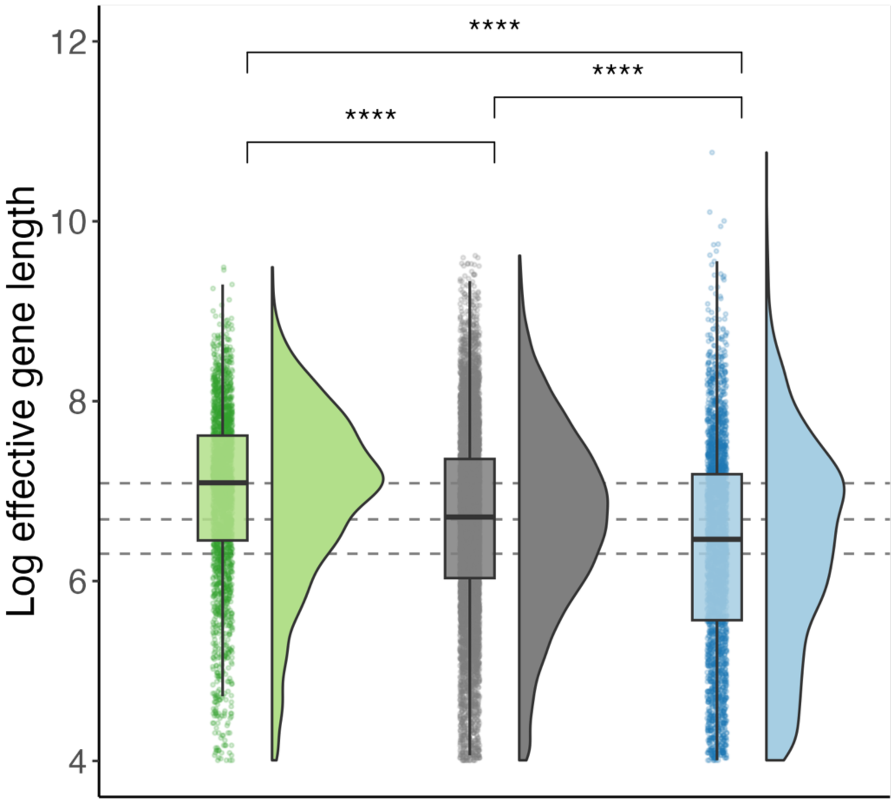

Figure S4. Log(10) effective length of *T. carbonaria* genes, grouped by caste-biased gene expression (left, green: queen-biased; middle, grey: caste-unbiased; right, blue: worker-biased). Significance values indicated by asterisks are the result of two-sided Wilcoxon tests following correction for multiple comparisons, and dashed lines represent median values of the three sets of genes.

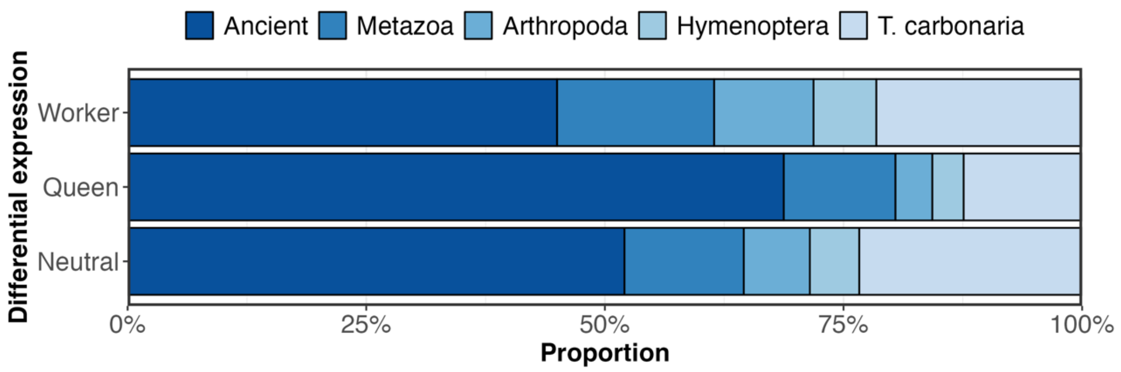

Figure S5. Proportion of *T. carbonaria* genes grouped by caste-biased gene expression (above: worker-biased; middle: queen-biased; bottom: caste-unbiased) belonging to different phylostrata.

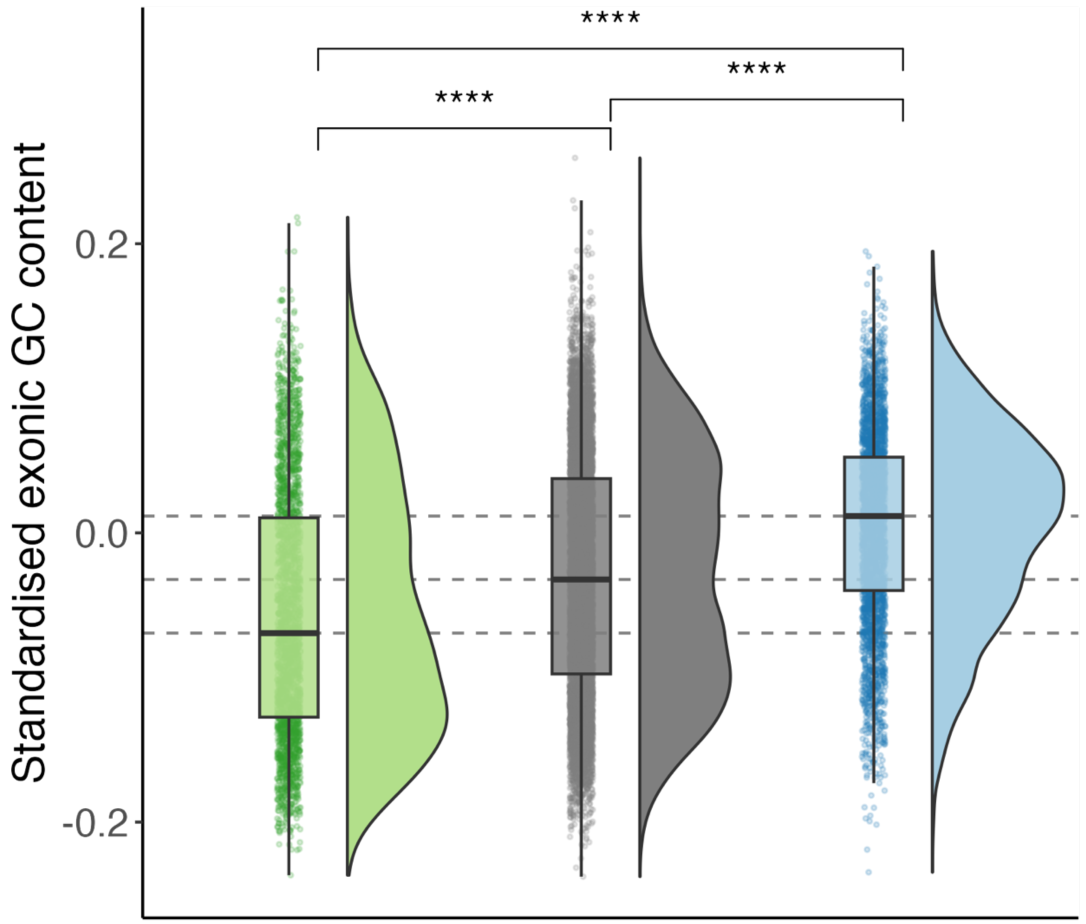

Figure S6. Scaled and zero-centered exonic GC content of *T. carbonaria* genes, grouped by caste-biased gene expression (left, green: queen-biased; middle, grey: caste-unbiased; right, blue: worker-biased). Significance values indicated by asterisks are the result of two-sided Wilcoxon tests following correction for multiple comparisons, and dashed lines represent median values of the three sets of genes.
